# A putative antiviral role of plant cytidine deaminases

**DOI:** 10.12688/f1000research.11111.2

**Published:** 2017-06-15

**Authors:** Susana Martín, José M. Cuevas, Ana Grande-Pérez, Santiago F. Elena

**Affiliations:** 1 Instituto de Biología Molecular y Celular de Plantas (IBMCP), CSIC-Universidad Politécnica de València, Campus UPV CPI 8E, Ingeniero Fausto Elio s/n, 46022 València, Spain; 2 Instituto de Biología Integrativa de Sistemas (I2SysBio), CSIC-Universitat de València, Parc Científic UV, Catedrático Agustín Escardino 9, 46980 Paterna, València, Spain; 3 Instituto de Hortofruticultura Subtropical y Mediterránea “La Mayora”, CSIC-Universidad de Málaga, Campus de Teatinos, 29071 Málaga, Spain; 4 Área de Genética, Universidad de Málaga, Campus de Teatinos, 29071 Málaga, Spain; 5 The Santa Fe Institute, 1399 Hyde Park Road, Santa Fe, NM, 87501, USA

**Keywords:** antiviral innate immunity, Cauliflower mosaic virus, error catastrophe, hypermutagenesis, mutational spectrum, plant-virus interaction, pararetrovirus, virus evolution

## Abstract

Background: A mechanism of innate antiviral immunity operating against viruses infecting mammalian cells has been described during the last decade.  Host cytidine deaminases ( *e.g*., APOBEC3 proteins) edit viral genomes, giving rise to hypermutated nonfunctional viruses; consequently, viral fitness is reduced through lethal mutagenesis.  By contrast, sub-lethal hypermutagenesis may contribute to virus evolvability by increasing population diversity.  To prevent genome editing, some viruses have evolved proteins that mediate APOBEC3 degradation.  The model plant *Arabidopsis thaliana* genome encodes nine cytidine deaminases ( *At*CDAs), raising the question of whether deamination is an antiviral mechanism in plants as well.

Methods: Here we tested the effects of expression of *At*CDAs on the pararetrovirus Cauliflower mosaic virus (CaMV). Two different experiments were carried out. First, we transiently overexpressed each one of the nine *A. thaliana* *AtCDA* genes in *Nicotiana* *bigelovii* plants infected with CaMV, and characterized the resulting mutational spectra, comparing them with those generated under normal conditions.  Secondly, we created *A. thaliana* transgenic plants expressing an artificial microRNA designed to knock-out the expression of up to six *AtCDA* genes.  This and control plants were then infected with CaMV.  Virus accumulation and mutational spectra where characterized in both types of plants.

Results:  We have shown that the *A. thaliana* *AtCDA1* gene product exerts a mutagenic activity, significantly increasing the number of G to A mutations *in vivo*, with a concomitant reduction in the amount of CaMV genomes accumulated.  Furthermore, the magnitude of this mutagenic effect on CaMV accumulation is positively correlated with the level of *AtCDA1* mRNA expression in the plant.

Conclusions: Our results suggest that deamination of viral genomes may also work as an antiviral mechanism in plants.

## Introduction

The human APOBEC (apolipoprotein B mRNA editing catalytic polypeptide-like) family includes enzymes that catalyze the hydrolytic deamination of cytidine to uridine or deoxycytidine to deoxyuridine. This family is composed of eleven known members: APOBEC1, APOBEC2, APOBEC3 (further classified as A3A to A3H), APOBEC4, and AID (activation induced deaminase). APOBEC proteins are associated with several functions involving editing of DNA or RNA (reviewed by Smith *et al* ^1^). APOBEC1 mediates deamination of cytidine at position 6666 of apolipoprotein B mRNA, resulting in the introduction of a premature stop codon and the production of the short form of the protein ^2– 4^. APOBEC2 is essential for muscle tissue development ^5^. APOBEC4 has no ascribed function so far ^6^. AID deaminates genomic ssDNA of B cells, initiating immunoglobulin somatic hypermutation and class switch processes ^7– 9^. Most notably, APOBEC3 enzymes participate in innate immunity against retroviruses and endogenous retroelements ^10– 12^. Sheehy *et al.* demonstrated that A3G also plays a role in immunity against human immunodeficiency virus type 1 (HIV-1) ^13^. For its antiviral role, A3G is packaged along with viral RNA ^14^. Upon infection of target cells and during the reverse transcription process, A3G deaminates the cytosine residues of the nascent first retroviral DNA strand into uraciles. The resulting uracil residues serve as templates for the incorporation of adenine, which at the end result in strand-specific C/G to T/A transitions and loss of infectivity through lethal mutagenesis ^15– 19^. On the other hand, sub-lethal mutagenic activity of APOBEC3 proteins may end up being an additional source for HIV-1 genetic diversity, hence bolstering its evolvability ^20– 22^. APOBEC3 proteins have been shown to inhibit other retroviruses (simian immunodeficiency virus ^23^, equine infectious anemia virus ^24^, foamy virus ^25^, human T-cell leukemia virus ^26^, and murine leukemia virus ^27^), pararetroviruses (hepatitis B virus ^28^) and DNA viruses (herpes simplex virus 1 ^29, 30^, Epstein-Barr virus ^30^, HSV-1 and EBV respectively, and human papillomavirus ^31^). In the cases of HSV-1 and EBV, the antiviral role of deaminases has not yet been demonstrated ^30^. Evidence also exists that A3G significantly interferes with negative-sense RNA viruses lacking a DNA replicative phase ^32^. For example, the transcription and protein accumulation of measles virus, mumps virus and respiratory syncytial virus (RSV) was reduced 50–70%, whereas the frequency of C/G to U/A mutations was ~4-fold increased after overexpressing A3G in Vero cells ^32^. In contrast, A3G plays no antiviral activity against influenza A virus despite being highly induced in infected cells as part of a general IFN-β response to infection ^33, 34^.

Human APOBEC belongs to a superfamily of polynucleotide cytidine and deoxycytidine deaminases distributed throughout the biological world ^35^. All family members contain a zinc finger domain (CDD), identifiable by the signature (H/C)-x-E-x25-30P-C-x-x-C. Plants are not an exception and, for example, the *Arabidopsis thaliana* genome encodes nine putative cytidine deaminases (with genes named *AtCDA1* to *AtCDA9*). Whilst the *AtCDA1* gene is located in chromosome II, the other eight genes are located in chromosome IV. In the case of rice and other monocots, only one CDA has been identified ^35^. Interestingly, this CDA expression was highly induced as part of the general stress response of rice against infection of the fungal pathogen *Magnaporthe grisea*, resulting in an excess of A to G and U to C mutations in defense-related genes ^36^. Edited dsRNAs might be retained in the nucleus and degraded, generating miRNAs and siRNAs ^37^. Given the relevance of deamination as an antiviral innate response in animals, we sought first to determine whether any of the *At*CDA proteins encoded by plants can participate in deaminating the genome of the pararetrovirus, cauliflower mosaic virus (CaMV; genus *Caulimovirus*, family *Caulimoviridae*) and, second, we sought to explore whether this deamination may negatively impact viral infection. We hypothesize that deamination may take place mainly at the reverse transcription step. The CaMV genome is constituted by a single molecule of circular double-stranded DNA of 8 kbp ^38^. The DNA of CaMV has three discontinuities, Δ1 in the negative-sense strand (or *a* strand), and Δ2 and Δ3 in the positive-sense strand (yielding the *b* and *g* strands). In short, the replication cycle of CaMV is as follows ^38^: in the nucleus of the infected cell, the *a* strand is transcribed into 35S RNA, with terminal repeats, that migrates to the cytoplasm. Priming of the 35S RNA occurs by the annealing of the 3’ end of tRNA ^met^ to the primer-binding site (PBS) sequence, leading to the synthesis of the DNA *a* strand by the virus’ reverse transcriptase. Then, the RNA in the heteroduplex is degraded by the virus’ RNaseH activity, leaving purine-rich regions that act as primers for the synthesis of the positive-sense DNA *b* and *g* strands.

Our results show that *AtCDA1* significantly increases the number of G to A mutations *in vivo*, and that there is a negative correlation between the amount of *AtCDA1* mRNA present in the cell and the load reached by CaMV, suggesting that deamination of viral genomes may also constitute a significant antiviral mechanism in plants.

## Methods

### Transient overexpression of *At*CDAs in *Nicotiana bigelovii* plants infected with CaMV

*AtCDA*s cDNAs were cloned under the 35S promoter in a pBIN61 vector ^39^. *N. bigelovii* plants were inoculated with CaMV virions purified from *Brassica rapa* plants ^40^ previously infected with the clone pCaMVW260 ^41^. *N. bigelovii* was chosen for this particular experiment for practical reasons: it is susceptible to CaMV infection, while *Nicotiana benthamiana* is not, and it is easily agroinfiltrated. Three symptomatic leafs were agroinfiltrated ^39^ with one of the nine *AtCDA*s and with the empty vector pBIN61, each on one half of the leaf. Samples were collected three days post-agroinfiltration.

### Inducible co-suppression of multiple *At*CDAs by RNAi

The design and cloning of the artificial micro-RNA (amiR) able to simultaneously suppress the expression of *At*CDAs 1, 2, 3, 4, 7, and 8 was performed as described in ref. 42. The amiRNA was cloned under the control of *Aspergillus nidulans* ethanol regulon ^43, 44^ and used to transform *A. thaliana* by the floral dip method ^45^. By doing so, we obtained the transgenic line amiR1-6-3. One-month-old seedlings of transgenic and wild-type *A. thaliana* were treated with 2% ethanol (or water for the control groups) three times every four days. Three days after the third treatment, plants were inoculated with the infectious clone pCaMVW260 as described in ref. 41. Infections were established by applying 1.31×10 ^11^ molecules of pCaMVW260 to each of three leaves per plant. Subsequently, plants were subjected to two additional treatments with 2% ethanol (or water) one and five days post-infection. Finally, samples were taken eight days after inoculation and handled as previously described ^46^. For each genotype (transgenic or wild-type) and treatment (ethanol or water) combination, 22 plants were analyzed.

### Detection of A/T enriched genomes

CaMV genomic DNA was purified using DNeasy Plant Mini Kit (Qiagen) according to manufacturer’s instructions. For detection of edited genomes 3D-PCR was performed using primers HCa8Fdeg and HCa8Rdeg. PCRs were performed in a Mastercycler ^®^ (Eppendorf) at denaturation temperatures 82.1°C, 82.9°C, 83.9°C, and 85.0°C. The 229 nt long PCR products obtained with the lowest denaturation temperature were cloned in pUC19 vector (Fermentas), transformed in *Escherichia coli* DH5α and sent to GenoScreen (Lille, France) for sequencing.

### RT-qPCR analysis of *AtCDA1* mRNA and qPCR analysis of CaMV load in transgenic plants

Total RNA was extracted from *A. thaliana* plants using the RNeasy ^®^ Plant Mini Kit (Qiagen), according to manufacturer’s instructions. *AtCDA1* specific primers qCDA1-F and qCDA1-R were designed using Primer Express software (Applied Biosystems). RT-qPCR reactions were performed using the One Step SYBR PrimeScript RT-PCR Kit II (Takara). Amplification, data acquisition and analysis were carried out using an Applied Biosystems Prism 7500 sequence detection system. All quantifications were performed using the standard curve method. To quantify *AtCDA1* mRNA, a full-ORF runoff transcript was synthetized with T7 RNA polymerase (Roche) using as template a PCR product obtained from cloned *AtCDA1* and primers T7-CDA1F and qCDA1-R. CaMV qPCR quantitation was performed as described in ref. 46.

### Primers

All primers used are listed in Supplementary Table S3.

## Results

### Effect of *At*CDAs overexpression on CaMV mutational spectrum

To test the mutagenic activity of *A. thaliana* CDAs, nine *N. bigelovii* plants were inoculated with CaMV. After systemic infection was established, we performed transient *AtCDA* overexpression experiments. To do so, the same leaf was agroinfiltrated twice; one half of the leaf was infiltrated with one of the nine *AtCDA* genes and the other half of the leaf was infiltrated with the empty vector. This test was done for all nine *AtCDA* genes in different plants. The presence of *AtCDA* mRNAs was verified by RT-PCR from DNase-treated RNA extracts. DNA was extracted from agroinfiltrated areas for 3D-PCR amplification of a 229 bp fragment in the ORF VII of CaMV. 3D-PCR uses a gradient of low denaturation temperatures during PCR to identify the lowest one, which potentially allows differential amplification of A/T rich hypermutated genomes ^47^. There were no differences in the lowest denaturation temperature that could result in differential amplification of controls and the *AtCDA*-agroinfiltrated samples, suggesting that hypermutated genomes should be at low frequency, if present at all.

**Figure 1.**
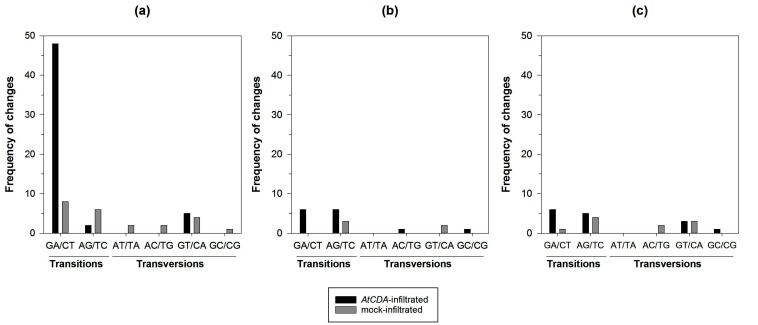
Number of mutations in CaMV genomes isolated from plant tissues agroinfiltrated with different *AtCDA*s. ( **a**) *AtCDA1*, ( **b**) *AtCDA2* and ( **c**) *AtCDA9*. The pBIN61 empty vector was agroinfiltrated in the same leaves than their corresponding *At*CDAs (mock). For each sample 20,034 nucleotides were sequenced.

PCR products obtained at the lowest denaturation temperature were cloned and sequenced. In a preliminary experiment, we sequenced 25 clones from each *AtCDA*/negative control pair ( Supplementary Table S1). At least one G to A transition was detected in clones from areas infiltrated with *AtCDA1*, *AtCDA2* and *AtCDA9* genes. For these three genes, we further increased the number of sequenced clones up to 106. The CaMV mutant spectra was significantly different between plants overexpressing *AtCDA1* and their respective negative controls ( Figure 1a: *χ* ^2^ = 25.760, 7 d.f., *P* = 0.001). This difference was entirely driven by the 471.43% increase in G to A transitions observed in the plants overexpressing *AtCDA1*. A thorough inspection of alignments showed that most of the G to A mutations (65.6%) detected in the different samples were located at the nucleotide position 181 ( Supplementary Table S1). By contrast, no overall difference existed between the mutant spectra of CaMV populations replicating in plants overexpressing *AtCDA2* ( Figure 1b: *χ* ^2^ = 8.944, 6 d.f., *P* = 0.177) or *AtCDA9* ( Figure 1c: *χ* ^2^ = 6.539, 8 d.f., *P* = 0.587) and their respective controls. Consistently, the mutant spectra from the three *AtCDA*-overexpressed samples were significantly heterogeneous ( *χ* ^2^ = 41.063, 16 d.f., *P* = 0.001), again due to the enrichment in G to A transitions observed in the case of *AtCDA1*. By contrast, the three independent control inoculation experiments showed homogeneous mutant spectra for CaMV ( *χ* ^2^ = 14.605, 18 d.f., *P* = 0.689), undistinguishable from the mutant spectra previously reported for natural isolates of this virus ^48^. The consistency of the mutant spectra observed for the three control experiments and with the spectrum described for a natural isolate of the virus suggests that under the physiological expression level of *AtCDA1*, the CaMV mutant spectrum is rather stable.

We conclude that overexpressing the *AtCDA1* gene results in a significant shift in CaMV genome composition towards G to A mutations, as expected from cytidine deaminase hypermutagenic activity.

### Effect of suppressing *AtCDA* expression on the viral load and mutational spectrum of CaMV

To test the effects of suppressing the expression of *AtCDA* on viral accumulation we produced a transgenic line of *A. thaliana* Col-0, named amiR1-6-3. This line was stably transformed with an amiR, controlled by the *A. nidulans* ethanol regulon to achieve ethanol-triggered RNAi-mediated simultaneous suppression of *AtCDA*s 1, 2, 3, 4, 7, and 8 expression. Transgenic and wild-type plants were subjected to periodical treatment with 2% ethanol (or water for the control groups). Subsequently, plants were inoculated with the infectious clone pCaMVW260 that expresses the genome of CaMV. Samples were taken eight days after inoculation and *AtCDA1* mRNA and CaMV viral load were quantified by real time RT-qPCR and qPCR, respectively, in the same samples. For each genotype and/or treatment, 22 plants were analyzed.

**Figure 2.**
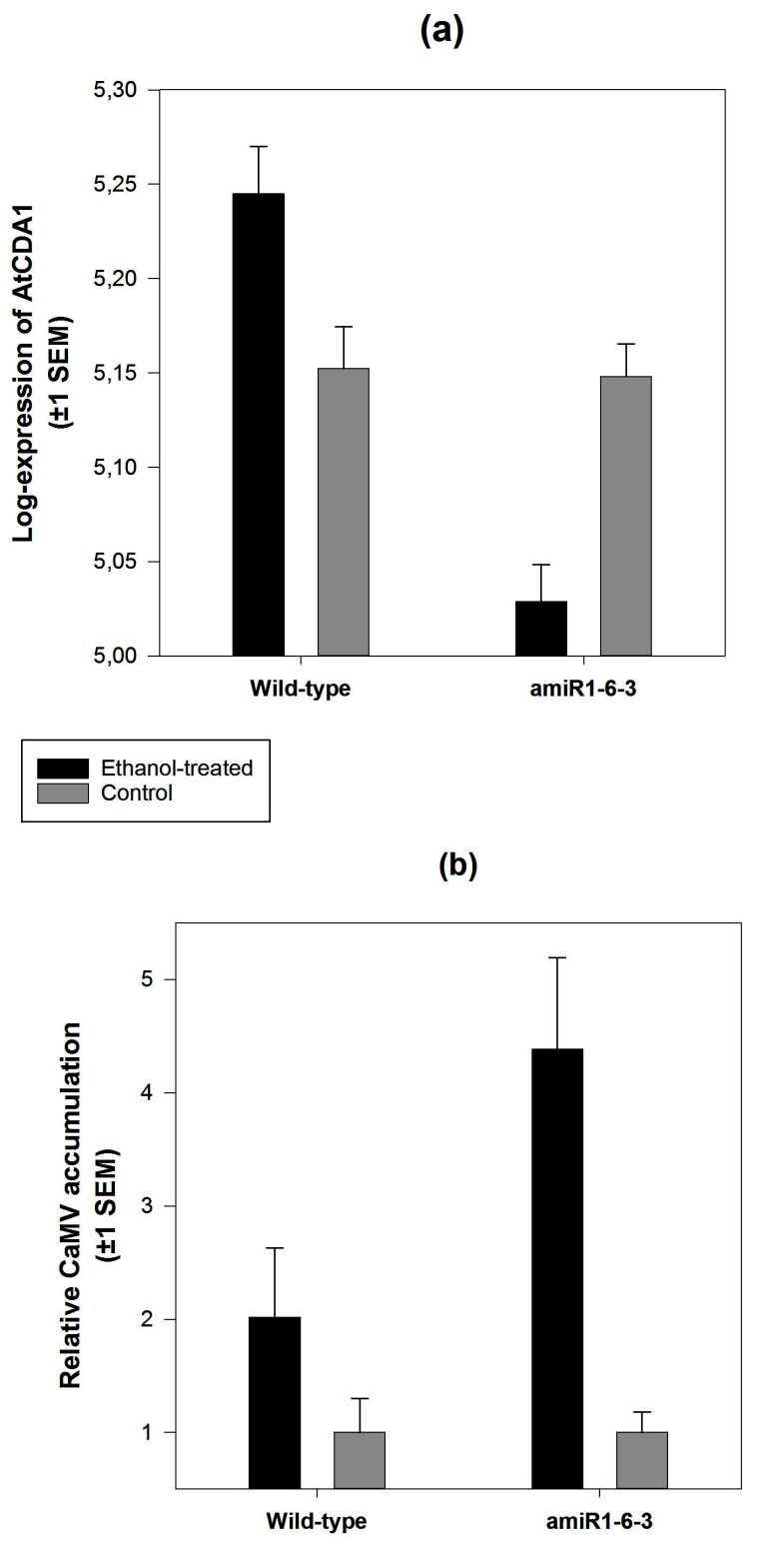
Accumulation of *AtCDA1* mRNA molecules and CaMV genomes. ( **a**) Number of *AtCDA1* mRNA molecules/80 ng total RNA quantified by RT-qPCR using the standard curve method for absolute quantification. ( **b**) Number of CaMV genomes/80 ng total DNA. For each block of plants (wild-type and amiR1-6-3), values were normalized to the average number of genomes estimated in the corresponding water-treated (control) plants.

The expression of *AtCDA1* mRNA depended on the plant genotype ( Figure 2a; GLM: • ^2^ = 28.085, 1 d.f., *P* < 0.001) as well as on the interaction of plant genotype and treatment (• ^2^ = 26.037, 1 d.f., *P* < 0.001), suggesting a differential accumulation of *AtCDA1* mRNA on each plant genotype depending on the amiR1-6-3 induction state. Ethanol treatment reduced the amount of *AtCDA1* mRNA by 24.01% in transgenic plants, proving that triggering the expression of the amiR1-6-3 significantly and efficiently silences the expression of *AtCDA1*. Unexpectedly, the effect was the opposite in wild-type plants, for which we observed 23.76% increase in *AtCDA1* mRNA accumulation ( Figure 2a) upon treatment with ethanol. This increase in expression of *AtCDA1* in wild-type plants after ethanol treatment and the underlying mechanisms certainly deserve to be investigated further. However, for the purpose of this study, its relevance is that it may increase the number of G to A mutations in the CaMV genome, thus making the antiviral effect stronger to some extent.

More interestingly, the relative accumulation of CaMV in ethanol-treated plants was significantly different, depending on the plant genotype being infected ( Figure 2b; Mann-Whitney *U* test, *P* = 0.002): silencing the *AtCDA1* gene bolstered CaMV accumulation to 103.10% compared to the accumulation observed in wild-type plants. Furthermore, there was a significant negative correlation between the number of molecules of *AtCDA1* mRNA and viral load (partial correlation coefficient controlling for treatment: *r* = –0.299, 86 d.f., *P* = 0.005).

**Figure 3.**
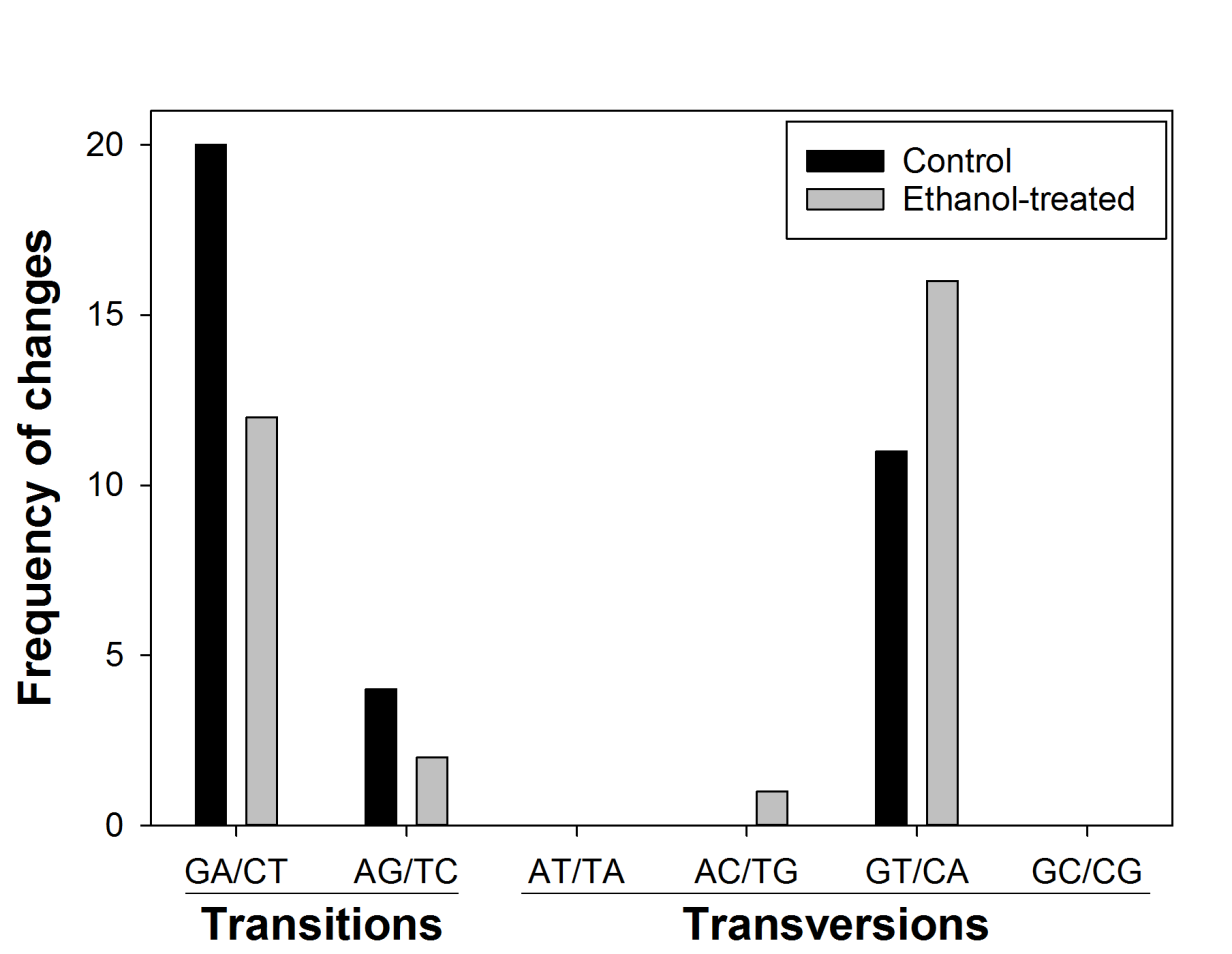
Number of mutations found pooling the CaMV sequences from ethanol-treated and control amiR1-6-3 plants (3 replicates). The number of nucleotides sequenced was 23,436 for control and 24,003 for ethanol-treated plants. Ethanol-treated plants turn on the expression of amiR1-6-3 that was designed to silence the expression of the *AtCDA1* gene.

Given the significant increase of viral load in plants with lower levels of *AtCDA1* mRNA, we sought the molecular signature of deamination in transgenic plants. For this, we selected three biological replicates from each treatment group (ethanol or control) and sequenced between 39–45 clones of the CaMV fragment from each replicate. As shown in Figure 3, silencing of the *AtCDA1* gene affects the composition of CaMV mutant spectrum by reducing the number of G to A transitions by 69.23%. Nevertheless, overall, both mutational spectra were not significantly different ( Figure 3: *χ* ^2^ = 9.108, 6 d.f., *P* = 0.168), prompting caution against making a definite conclusion on the role of deamination in the observed increase in CaMV accumulation.

We conclude that suppressing the expression of the *AtCDA*s 1, 2, 3, 4, 7, and 8 significantly reduces the accumulation of CaMV. However, the characterization of the mutant spectrum of the same CaMV populations provides no strong enough support to the cytidine deamination hypothesis.

## Discussion

Lethal mutagenesis through deamination of RNA/DNA by cytidine deaminases has been proven to work as an antiviral mechanism against retroviruses ^16– 19, 23– 27^, and some DNA ^28– 31^ and RNA ^32^ viruses infecting mammals. Our results show that the *A. thaliana CDA1* gene has some degree of mutagenic activity on the pararetrovirus CaMV genome. Moreover, simultaneously suppressing the expression of a subset of *AtCDA*s, including *AtCDA1*, increased CaMV load, strongly suggesting an antiviral role for *At*CDAs. This role of AtCDA1 is congruent with the very recent observation by Chen *et al.* that only the product of *AtCDA1* is required for *in vivo* homeostasis of pyrimidines while the other eight members of the gene family may be pseudogenes ^49^

Our data show that *At*CDAs probably restrict CaMV replication through a process similar to the restriction of HIV-1 by APOBEC3. CaMV replicates in the cytoplasm by reverse transcription using the positive-sense 35S RNA as template. As for HIV-1, the first strand negative-sense cDNA could be deaminated during reverse transcription, transforming deoxycytidine into deoxyuridine. Then, when the positive-sense strand is produced, an A is incorporated instead of a G, increasing the proportion of G to A mutations. In the case of HIV-1, this G to A mutational bias is explained by A3G and A3H specificity for single negative stranded DNA: during HIV-1 replication, C to G transitions are rare and restricted to the PBS site and U3 regions in the 5’ long terminal repeat, where positive-stranded DNA is predicted to become transiently single stranded ^50^. Similarly, during CaMV replication the negative strand remains single stranded, while the positive is copied from it and remains double stranded ^51^. Surprisingly, for *AtCDA1,* C to T mutations were also increased; the region studied here is close to the 5’ end of CaMV, which contains the PBS for negative-strand synthesis and the ssDNA discontinuity ∆1. The observed C to T transitions could reflect transient positive-stranded ssDNA in the 5’ terminal region during reverse transcription, nevertheless a different substrate specificity of *A. thaliana* CDAs cannot be ruled out.

Evidences from studies with different mammalian viruses suggest that APOBEC enzymes may have an antiviral role not only against DNA viruses and retroviruses but also against some RNA viruses ^32^. Our evidences for a potential antiviral role of a plant CDA is restricted to the case of a pararetrovirus and thus the question is whether this mechanism would also operate against other types of plant viruses. Lin *et al.* described the spectrum of mutations accumulated in a non-coding sequence artificially inserted in the genome of turnip mosaic virus (TuMV), a prototypical RNA plant virus, during infection of *N. benthamiana* plants ^52^. C to A and C to U transitions were significantly over represented in the mutant spectrum, and the authors already suggested this bias was compatible with TuMV genome being edited by CDA enzymes ^52^.

Most of the G to A transitions detected in agroinfiltration experiments were located in the G at position 181. HIV-1 hypermutated genomes show mutational hot spots as well, which are due to preference of A3G and A3F for deamination of the third C in 5’-CCC (negative-strand) and 5’-GGC, respectively ^53, 54^. The sequence context of the C complementary to G181 (5’-GGC) differs from what has been described for APOBEC3 as hotspot for deamination, suggesting that if *At*CDAs had a context preference, it would be different from the one described for A3G. However, given the low number of mutations found, we should be cautious when concluding whether *At*CDAs have a possible sequence-context preference. Since our experiments were performed *in vivo*, negative selection is expected to purge genomes carrying deleterious mutations. To explore this possibility, we have checked the consequence of mutations in the protein encoded by the ORF VII ( Supplementary Table S1) for the case of plant agroinfiltrated with *AtCDA1* and its corresponding paired control . Eight out of the 22 different mutations observed in CaMV populations replicating in presence of *AtCDA1* were nonsynonymous, thus in agreement with previous observations that most G to A transitions in CaMV are synonymous ^54^. Two remarks can be made about these numbers. First, quite surprisingly, six of these eight nonsynonymous mutations resulted in stop codons affecting two different positions (amino acids C58 and Y71). Second, transition G181A is synonymous. For CaMV replicating in the corresponding control half-leaf (agroinfiltrated with the empty pBIN61 vector), three out of the 14 different mutations observed were nonsynonymous, one of them also resulting in an stop in codon 58. No significant differences exist among the relative ratio of nonsynonymous to synonymous mutations in both samples (Fisher’s exact test *P* = 0.467). Despite the mutagenic effect of *At*CDA1 over the CaMV population, the number of nonsynonymous mutations relative to the number of synonymous mutations is not altered, thus suggesting negative selection works, at least, as efficiently as it does in the control population. The same conclusion is reached if we only focus the comparison in the number of nonsynonymous mutations resulting in stop codons. This potential purifying effect of selection could account for our failure to detect largely hypermutated genomes, and demonstrates the need for developing new selection-free assays to further characterize *At*CDA-induced mutagenesis. Despite the apparent low number of deamination mutations observed, it has a significant impact in CaMV accumulation ( Figure 2b), thus suggesting that a low threshold of G to A transition bias may be enough to lead to a reduction in viral load.

Although there is not a demonstrated correlation between the expression of APOBEC3 and mutational bias of viruses infecting mammals, caulimoviruses have an excess of G to A transitions in synonymous positions ^55^. In *A. thaliana* plants, we found that silencing of *AtCDA1* reduced the frequency of G to A transitions in the CaMV genome, suggesting a contribution of *At*CDAs to the nucleotide bias found in caulimoviruses. The increased viral load in CDA-silenced *A. thaliana* plants strongly suggests that deamination of viral genomes may work as an antiviral mechanism in plants, leading to questions about how general this mechanism might be, and how it may contribute to viral evolution. Describing a new natural antiviral mechanism in plants opens new research avenues for the development of new durable control strategies.

## Data availability

The data referenced by this article are under copyright with the following copyright statement: Copyright: © 2017 Martín S et al.

Data associated with the article are available under the terms of the Creative Commons Zero "No rights reserved" data waiver (CC0 1.0 Public domain dedication). 

All datasets that support the findings in this study are available at LabArchives with DOI: 10.6070/H4TD9VD5.

‘File Sequence_data_for_Figure_1.zip’ contains the FASTA files with the sequence data used to generate the mutational spectra shown in Figure 1. 

‘Data_for_Figure_2a.xlsx’ contains the *AtCDA1* expression data used to generate Figure 2a. 

‘Data_for_Figure_2b.xlsx’ contains the CaMV accumulation data used to generate Figure 2b. 

‘Sequence_data_for_Figure_3.zip’ contains the FASTA files with sequence data used to generate the mutational spectra shown in Figure 3. 

## Supplementary material

**Supplementary Table S1. Nucleotide substitutions detected in the overexpression experiments.** For each of the nine infiltrated plants, the substitutions observed in the clonal sequences analyzed at the overexpressed ( *AtCDA1* to *AtCDA9*) and control (pBIN61-infiltrated) regions are shown. In some cases, a given substitution is present in several clonal sequences from the same sample and the number of times it appears is indicated between parentheses. G to A transitions are shaded in grey. Nucleotide positions are given according to CaMV isolate W260, GenBank accession JF809616.1.

.

**Supplementary Table S2. Nucleotide substitutions found in *A. thaliana* transgenic plants with or without inducing the expression of amiR3-1-9 that silences the expression of several AtCDAs.** In some cases, a given substitution is present in several clonal sequences from the same sample and the number of times it appears is indicated between brackets. G to A transitions are shaded in grey. Nucleotide positions are given according to CaMV isolate W260, GenBank accession JF809616.1.

.

**Supplementary Table S3. Primers used in this study.**

## References

[1] Smith HC Bennett RP Kizilyer A: Functions and regulation of the APOBEC family of proteins. *Semin Cell Dev Biol.* 2012;23(3):258–268. 10.1016/j.semcdb.2011.10.004 22001110 PMC4017262

[2] Driscoll DM Zhang Q: Expression and characterization of p27, the catalytic subunit of the apolipoprotein B mRNA editing enzyme. *J Biol Chem.* 1994;269(31):19843–19847. 8051066

[3] Navaratnam N Morrison JR Bhattacharya S: The p27 catalytic subunit of the apolipoprotein B mRNA editing enzyme is a cytidine deaminase. *J Biol Chem.* 1993;268(28):20709–20712. 8407891

[4] Teng B Burant CF Davidson NO: Molecular cloning of an apolipoprotein B messenger RNA editing protein. *Science.* 1993;260(5115):1816–1819. 10.1126/science.8511591 8511591

[5] Sato Y Probst HC Tatsumi R: Deficiency in APOBEC2 leads to a shift in muscle fiber type, diminished body mass, and myopathy. *J Biol Chem.* 2010;285(10):7111–7118. 10.1074/jbc.M109.052977 20022958 PMC2844160

[6] Rogozin IB Basu MK Jordan IK: APOBEC4, a new member of the AID/APOBEC family of polynucleotide (deoxy)cytidine deaminases predicted by computational analysis. *Cell Cycle.* 2005;4(9):1281–1285. 10.4161/cc.4.9.1994 16082223

[7] Muramatsu M Sankaranand VS Anant S: Specific expression of activation-induced cytidine deaminase (AID), a novel member of the RNA-editing deaminase family in germinal center B cells. *J Biol Chem.* 1999;274(26):18470–18476. 10.1074/jbc.274.26.18470 10373455

[8] Arakawa H Hauschild J Buerstedde JM: Requirement of the activation-induced deaminase (AID) gene for immunoglobulin gene conversion. *Science.* 2002;295(5558):1301–1306. 10.1126/science.1067308 11847344

[9] Fugmann SD Schatz DG: Immunology. One AID to unite them all. *Science.* 2002;295(5558):1244–1245. 10.1126/science.1070023 11847327

[10] Chiu YL Witkowska HE Hall SC: High-molecular-mass APOBEC3G complexes restrict *Alu* retrotransposition. *Proc Natl Acad Sci U S A.* 2006;103(42):15588–15593. 10.1073/pnas.0604524103 17030807 PMC1592537

[11] Schumann GG: APOBEC3 proteins: major players in intracellular defence against LINE-1-mediated retrotransposition. *Biochem Soc Trans.* 2007;35(Pt 3):637–642. 10.1042/BST0350637 17511669

[12] Esnault C Millet J Schwartz O: Dual inhibitory effects of APOBEC family proteins on retrotransposition of mammalian endogenous retroviruses. *Nucl Acids Res.* 2006;34(5):1522–1531. 10.1093/nar/gkl054 16537839 PMC1401513

[13] Sheehy AM Gaddis NC Choi JD: Isolation of a human gene that inhibits HIV-1 infection and is suppressed by the viral Vif protein. *Nature.* 2002;418(6898):646–650. 10.1038/nature00939 12167863

[14] Smith HC: APOBEC3G: a double agent in defense. *Trends Biochem Sci.* 2011;36(5):239–244. 10.1016/j.tibs.2010.12.003 21239176 PMC3086942

[15] Mangeat B Turelli P Caron G: Broad antiretroviral defence by human APOBEC3G through lethal editing of nascent reverse transcripts. *Nature.* 2003;424(6944):99–103. 10.1038/nature01709 12808466

[16] Zhang H Yang B Pomerantz RJ: The cytidine deaminase CEM15 induces hypermutation in newly synthesized HIV-1 DNA. *Nature.* 2003;424(6944):94–98. 10.1038/nature01707 12808465 PMC1350966

[17] Browne EP Allers C Landau NR: Restriction of HIV-1 by APOBEC3G is cytidine deaminase-dependent. *Virology.* 2009;387(2):313–321. 10.1016/j.virol.2009.02.026 19304304 PMC3708462

[18] Miyagi E Opi S Takeuchi H: Enzymatically active APOBEC3G is required for efficient inhibition of human immunodeficiency virus type 1. *J Virol.* 2007;81(24):13346–13353. 10.1128/JVI.01361-07 17928335 PMC2168852

[19] Schumacher AJ Haché G Macduff DA: The DNA deaminase activity of human APOBEC3G is required for Ty1, MusD, and *human immunodeficiency virus* type 1 restriction. *J Virol.* 2008;82(6):2652–2660. 10.1128/JVI.02391-07 18184715 PMC2259018

[20] Sadler HA Stenglein MD Harris RS: APOBEC3G contributes to HIV-1 variation through sublethal mutagenesis. *J Virol.* 2010;84(14):7396–7404. 10.1128/JVI.00056-10 20463080 PMC2898230

[21] Mulder LC Harari A Simon V: Cytidine deamination induced HIV-1 drug resistance. *Proc Natl Acad Sci U S A.* 2008;105(14):5501–5506. 10.1073/pnas.0710190105 18391217 PMC2291111

[22] Russell RA Moore MD Hu WS: APOBEC3G induces a hypermutation gradient: purifying selection at multiple steps during HIV-1 replication results in levels of G-to-A mutations that are high in DNA, intermediate in cellular viral RNA, and low in virion RNA. *Retrovirology.* 2009;6:16. 10.1186/1742-4690-6-16 19216784 PMC2657108

[23] Hultquist JF Lengyel JA Refsland EW: Human and rhesus APOBEC3D, APOBEC3F, APOBEC3G, and APOBEC3H demonstrate a conserved capacity to restrict Vif-deficient HIV-1. *J Virol.* 2011;85(21):11220–11234. 10.1128/JVI.05238-11 21835787 PMC3194973

[24] Zielonka J Bravo IG Marino D: Restriction of *equine infectious anemia virus* by equine APOBEC3 cytidine deaminases. *J Virol.* 2009;83(15):7547–7559. 10.1128/JVI.00015-09 19458006 PMC2708611

[25] Delebecque F Suspène R Calattini S: Restriction of *foamy viruses* by APOBEC cytidine deaminases. *J Virol.* 2006;80(2):605–614. 10.1128/JVI.80.2.605-614.2006 16378963 PMC1346872

[26] Mahieux R Suspène R Delebecque F: Extensive editing of a small fraction of *Human T-cell leukemia virus* type 1 genomes by four APOBEC3 cytidine deaminases. *J Gen Virol.* 2005;86(Pt 9):2489–2494. 10.1099/vir.0.80973-0 16099907

[27] Dang Y Wang X Esselman WJ: Identification of APOBEC3DE as another antiretroviral factor from the human APOBEC family. *J Virol.* 2006;80(21):10522–10533. 10.1128/JVI.01123-06 16920826 PMC1641744

[28] Bonvin M Achermann F Greeve I: Interferon-inducible expression of APOBEC3 editing enzymes in human hepatocytes and inhibition of *hepatitis B virus* replication. *Hepatology.* 2006;43(6):1364–1374. 10.1002/hep.21187 16729314

[29] Gee P Ando Y Kitayama H: APOBEC1-mediated editing and attenuation of *Herpes simplex virus* 1 DNA indicate that neurons have an antiviral role during herpes simplex encephalitis. *J Virol.* 2011;85(19):9726–9736. 10.1128/JVI.05288-11 21775448 PMC3196441

[30] Suspène R Aynaud MM Koch S: Genetic editing of *herpes simplex virus* 1 and *Epstein-Barr herpesvirus* genomes by human APOBEC3 cytidine deaminases in culture and *in vivo*. *J Virol.* 2011;85(15):7594–7602. 10.1128/JVI.00290-11 21632763 PMC3147940

[31] Wang Z Wakae K Kitamura K: APOBEC3 deaminases induce hypermutation in human papillomavirus 16 DNA upon beta interferon stimulation. *J Virol.* 2014;88(2):1308–1317. 10.1128/JVI.03091-13 24227842 PMC3911654

[32] Fehrholz M Kendl S Prifert C: The innate antiviral factor APOBEC3G targets replication of measles, mumps and respiratory syncytial viruses. *J Gen Virol.* 2012;93(Pt 3):565–576. 10.1099/vir.0.038919-0 22170635

[33] Pauli EK Schmolke M Hofmann H: High level expression of the anti-retroviral protein APOBEC3G is induced by influenza A virus but does not confer antiviral activity. *Retrovirology.* 2009;6:38. 10.1186/1742-4690-6-38 19371434 PMC2672920

[34] Wang GF Lin SY Zhang H: Apobec 3F and apobec 3G have no inhibition and hypermutation effect on the human *influenza A virus*. *Acta Virol.* 2008;52(3):193–194. 18999897

[35] Conticello SG Thomas CJ Petersen-Mahrt SK: Evolution of the AID/APOBEC family of polynucleotide (deoxy)cytidine deaminases. *Mol Biol Evol.* 2005;22(2):367–377. 10.1093/molbev/msi026 15496550

[36] Gowda M Venu RC Li H: *Magnaporthe grisea* infection triggers RNA variation and antisense transcript expression in rice. *Plant Physiol.* 2007;144(1):524–533. 10.1104/pp.107.095653 17351054 PMC1913787

[37] Blow MJ Grocock RJ van Dongen S: RNA editing of human microRNAs. *Genome Biol.* 2006;7(4):R27. 10.1186/gb-2006-7-4-r27 16594986 PMC1557993

[38] Haas M Bureau M Geldreich A: *Cauliflower mosaic virus*: still in the news. *Mol Plant Pathol.* 2002;3(6):419–429. 10.1046/j.1364-3703.2002.00136.x 20569349

[39] Bendahmane A Querci M Kanyuka K: *Agrobacterium* transient expression system as a tool for the isolation of disease resistance genes: application to the *Rx2* locus in potato. *Plant J.* 2000;21(1):73–81. 10.1046/j.1365-313x.2000.00654.x 10652152

[40] Schoelz JE Shepherd RJ Daubert S: Region VI of *cauliflower mosaic virus* encodes a host range determinant. *Mol Cell Biol.* 1986;6(7):2632–2637. 10.1128/MCB.6.7.2632 3785205 PMC367819

[41] Scholelz JE Shepherd RJ: Host range control of *cauliflower mosaic virus*. *Virology.* 1988;162(1):30–37. 10.1016/0042-6822(88)90391-1 3341113

[42] Schwab R Ossowski S Riester M: Highly specific gene silencing by artificial microRNAs in *Arabidopsis*. *Plant Cell.* 2006;18(5):1121–1133. 10.1105/tpc.105.039834 16531494 PMC1456875

[43] Caddick MX Greenland AJ Jepson I: An ethanol inducible gene switch for plants used to manipulate carbon metabolism. *Nat Biotech.* 1998;16(2):177–180. 10.1038/nbt0298-177 9487526

[44] Roslan HA Salter MG Wood CD: Characterization of the ethanol-inducible *alc* gene-expression system in *Arabidopsis thaliana.* *Plant J.* 2001;28(2):225–235. 10.1046/j.1365-313X.2001.01146.x 11722766

[45] Clough SJ Bent AF: Floral dip: a simplified method for *Agrobacterium*-mediated transformation of *Arabidopsis thaliana*. *Plant J.* 1998;16(6):735–743. 10.1046/j.1365-313x.1998.00343.x 10069079

[46] Martín S Elena SF: Application of game theory to the interaction between plant viruses during mixed infections. *J Gen Virol.* 2009;90(Pt 11):2815–2820. 10.1099/vir.0.012351-0 19587130

[47] Suspène R Henry M Guillot S: Recovery of APOBEC3-edited *human immunodeficiency virus* G–>A hypermutants by differential DNA denaturation PCR. *J Gen Virol.* 2005;86(Pt 1):125–129. 10.1099/vir.0.80426-0 15604439

[48] Chenault KD Melcher U: Patterns of nucleotide sequence variation among *cauliflower mosaic virus* isolates. *Biochimie.* 1994;76(1):3–8. 10.1016/0300-9084(94)90056-6 8031902

[49] Chen M Herde M Witte CP: Of the Nine Cytidine Deaminase-Like Genes in Arabidopsis, Eight Are Pseudogenes and Only One Is Required to Maintain Pyrimidine Homeostasis *in Vivo*. *Plant Physiol.* 2016;171(2):799–809. 10.1104/pp.15.02031 27208239 PMC4902590

[50] Yu Q König R Pillai S: Single-strand specificity of APOBEC3G accounts for minus-strand deamination of the HIV genome. *Nat Struct Mol Biol.* 2004;11(5):435–42. 10.1038/nsmb758 15098018

[51] Marco Y Howell SH: Intracellular forms of viral DNA consistent with a model of reverse transcriptional replication of the *cauliflower mosaic virus* genome. *Nucl Acids Res.* 1984;12(3):1517–1528. 10.1093/nar/12.3.1517 6199741 PMC318593

[52] Lin SS Wu HW Elena SF: Molecular evolution of a viral non-coding sequence under the selective pressure of amiRNA-mediated silencing. *PLoS Pathog.* 2009;5(2):e1000312. 10.1371/journal.ppat.1000312 19247440 PMC2642722

[53] Liddament MT Brown WL Schumacher AJ: APOBEC3F properties and hypermutation preferences indicate activity against HIV-1 *in vivo.* *Curr Biol.* 2004;14(15):1385–91. 10.1016/j.cub.2004.06.050 15296757

[54] Kohli RM Maul RW Guminski AF: Local sequence targeting in the AID/APOBEC family differentially impacts retroviral restriction and antibody diversification. *J Biol Chem.* 2010;282(52):40956–40964. 10.1074/jbc.M110.177402 20929867 PMC3003395

[55] Müller V Bonhoeffer S: Guanine-adenine bias: a general property of retroid viruses that is unrelated to host-induced hypermutation. *Trends Genet.* 2005;21(5):264–268. 10.1016/j.tig.2005.03.004 15851060

