# Supplementary material for: A putative antiviral role of plant cytidine deaminases
Source: F1000Res. 2017 Jun 15;6:622. Originally published 2017 May 3. [Version 2] doi: 10.12688/f1000research.11111.2 (PMC5461918; doi:10.12688/f1000research.11111.2)
Supplement: Supplementary file 1 [file f1000research-6-12856-s0001.tgz › a7ad727f-3253-4135-94d1-6a53ab32360e.docx]

| **Supplementary Table S1. Nucleotide substitutions detected in the overexpression experiments.** For each of the nine infiltrated plants, the substitutions observed in the clonal sequences analyzed at the overexpressed (*AtCDA1* to *AtCDA9*) and control (pBIN61-infiltrated) regions are shown. In some cases, a given substitution is present in several clonal sequences from the same sample and the number of times it appears is indicated between parentheses. G to A transitions are shaded in grey. Nucleotide positions are given according to CaMV isolate W260, GenBank accession JF809616.1. | | | | | | |
| --- | --- | --- | --- | --- | --- | --- |
| **Sample** | | **Number of clones** | **Mutation** | **Sample** | **Number of clones** | **Mutation** |
| *AtCDA1* | 106 | A91G | pBIN61 | 106 | T72C (2) |  |
|  |  | G129A |  |  | A84G |  |
|  |  | C131T |  |  | T98C |  |
|  |  | C132T |  |  | C135G (D45E) |  |
|  |  | C143T |  |  | A162T (2) |  |
|  |  | G148T |  |  | C174A (C58stop) |  |
|  |  | G148A |  |  | G181T |  |
|  |  | G153A |  |  | G181A (6) |  |
|  |  | C157A (2) (F52L) |  |  | C206T |  |
|  |  | G159A (Q53K) |  |  | G210A |  |
|  |  | C168T |  |  | A215G |  |
|  |  | G169A |  |  | C224A |  |
|  |  | C174A (C58stop) |  |  | A235G |  |
|  |  | G175A (C58stop) |  |  | G237T (K79E) |  |
|  |  | G181A (27) |  |  |  |  |
|  |  | G190A (3) |  |  |  |  |
|  |  | C199T |  |  |  |  |
|  |  | G210A |  |  |  |  |
|  |  | C213T (3) (Y71stop) |  |  |  |  |
|  |  | C213A (Y71stop) |  |  |  |  |
|  |  | G214A (2) (Y71stop) |  |  |  |  |
|  |  | A215G (Y71stop) |  |  |  |  |
| *AtCDA2* | 106 | T72C | pBIN61 | 106 | T80C |  |
|  |  | A77G |  |  | A161G |  |
|  |  | A84G |  |  | C196A |  |
|  |  | G126A |  |  | G221T |  |
|  |  | T154C |  |  | A232G |  |
|  |  | G169A |  |  |  |  |
|  |  | G181A (3) |  |  |  |  |
|  |  | C186G |  |  |  |  |
|  |  | A202C |  |  |  |  |
|  |  | T223C |  |  |  |  |
|  |  | G231A |  |  |  |  |
|  |  | A251G |  |  |  |  |
| *AtCDA3* | 24 | A101G | pBIN61 | 24 | A82G |  |
|  |  | C180T |  |  |  |  |
| *AtCDA4* | 24 | C125T | pBIN61 | 24 | G169T |  |
|  |  |  |  |  | T223C |  |
| *AtCDA5* | 26 | T79C | pBIN61 | 26 | A121G |  |
|  |  | C117T |  |  | A136G |  |
|  |  | T140A |  |  | C186A |  |
|  |  | C165G (3) |  |  | C189T |  |
|  |  |  |  |  | A212G |  |
| *AtCDA6* | 25 | C147A | pBIN61 | 25 | C135G |  |
|  |  | G224T |  |  |  |  |
| *AtCDA7* | 24 | T96C | pBIN61 | 24 | T193C |  |
|  |  | A103G |  |  |  |  |
|  |  | T140C |  |  |  |  |
|  |  | C186T |  |  |  |  |
| *AtCDA8* | 24 | T114C | pBIN61 | 24 | G68C |  |
|  |  | C157T (5) |  |  | T98C |  |
|  |  | T195C |  |  | G127C |  |
|  |  |  |  |  | G214T |  |
|  |  |  |  |  | T248C |  |
| *AtCDA9* | 106 | G85T | pBIN61 | 106 | T114C |  |
|  |  | C95T |  |  | T119C |  |
|  |  | G126A |  |  | T128C |  |
|  |  | T128C |  |  | C143T |  |
|  |  | C146A (2) |  |  | C147A |  |
|  |  | A161G |  |  | G153T |  |
|  |  | A163G |  |  | T187G |  |
|  |  | T176C |  |  | A212G |  |
|  |  | G181A (2) |  |  | G214A |  |
|  |  | G218C |  |  | G221T |  |
|  |  | G231A |  |  | A224C |  |
|  |  | A235G |  |  |  |  |
|  |  | C241T |  |  |  |  |
