# Supplementary material for: A putative antiviral role of plant cytidine deaminases
Source: F1000Res. 2017 Jun 15;6:622. Originally published 2017 May 3. [Version 2] doi: 10.12688/f1000research.11111.2 (PMC5461918; doi:10.12688/f1000research.11111.2)
Supplement: Supplementary file 2 [file f1000research-6-12856-s0002.tgz › bc4753bc-c5e8-4b8a-bade-48252d18f3ec.docx]

| **Supplementary Table S2. Nucleotide substitutions found in *A. thaliana* transgenic plants with or without inducing the expression of amiR3-1-9 that silences the expression of several *At*CDAs.** In some cases, a given substitution is present in several clonal sequences from the same sample and the number of times it appears is indicated between brackets. G to A transitions are shaded in grey. Nucleotide positions are given according to CaMV isolate W260, GenBank accession JF809616.1. | | |
| --- | --- | --- |
| **Sample** | **Number of of clones** | **Change** |
| Ethanol treated, plant 2 | 45 | G129T |
|  |  | C144T |
|  |  | C147A |
|  |  | G153A |
|  |  | G159T |
|  |  | C162T (2) |
|  |  | C165T (3) |
|  |  | C168A |
|  |  | G173A |
|  |  | G175T (2) |
|  |  | G175A |
|  |  | G181T |
|  |  | C182T |
|  |  | G190T (2) |
|  |  | G198T (2) |
|  |  | C199A |
|  |  | C206A (2) |
|  |  | G207T |
|  |  | G214A |
| Ethanol treated, plant 3 | 42 | C135T |
|  |  | C185T |
| Ethanol treated, plant 20 | 39 | T74C |
|  |  | A121G |
|  |  | G126T |
| Control, plant 16 | 40 | T72C |
|  |  | A232G |
| Control, plant 19 | 43 | A212G |
| Control, plant 21 | 41 | T115C |
|  |  | G127T |
|  |  | G133A |
|  |  | G133T |
|  |  | C143T |
|  |  | C144T |
|  |  | C147A |
|  |  | G152A |
|  |  | G153A |
|  |  | C165T |
|  |  | G173A |
|  |  | C174T |
|  |  | G175A (9) |
|  |  | G175T |
|  |  | G181T |
|  |  | C182A |
|  |  | G190T (2) |
|  |  | C196T |
|  |  | G198T |
|  |  | C201T |
|  |  | G204T |
|  |  | C206T |
