# Supplementary material for: A putative antiviral role of plant cytidine deaminases
Source: F1000Res. 2017 Jun 15;6:622. Originally published 2017 May 3. [Version 2] doi: 10.12688/f1000research.11111.2 (PMC5461918; doi:10.12688/f1000research.11111.2)
Supplement: Supplementary file 3 [file f1000research-6-12856-s0000.tgz › e4490a3c-e566-4750-a303-0df6c5fa2ecb.pdf]

Supplementary Table S3. Primers used in this study.

Primer Name	Sequence 5´→3’[a]	Template	Purpose
ORF-CDA1 F	ATGCG*GGATCC*ATGGATAAGCCAAGCTTCGTAATC	CDA1	
ORF-CDA1 R	GATCG*CCCCGGG*CTAAGCTTCATAGCAATGAAACAC	CDA1	Cloning of *At*CDA ORFs in the expression vector pBIN61
ORF-CDA2 F	ATGCG*GGATCC*ATGGCACAACGTCCTAACTTGTTA	CDA2	
ORF-CDA2 R	GATCG*CCCCGGG*TTAATAATGAAGACCAGTGTAATCC	CDA2	
ORF-CDA3 F	ATGCG*GGATCC*ATGGCACAAGATCAGTACAAGTTCG	CDA3	
ORF-CDA3 R	GATCGC*CCCGGG*TCATCCACGGTACCAGTTCCC	CDA3	
ORF-CDA4 F	ATGACACAACAGCTCAAATTCATTC	CDA4	
ORF-CDA4 R	TCAATTTTCTGCGGTCTTGTGACAA	CDA4	
ORF-CDA5 F	ATGCG*GGATCC*ATGGCACAACAGTACAAGTTCGTT	CDA5	
ORF-CDA5 R	GATCG*CCCCGGG*TTATAGTTTGATCCAATCTTTGAGCT	CDA5	
ORF-CDA6 F	GATCGC*CCCGGG*ATGAAGTTCGTTTACACTCCCAG	CDA6	
ORF-CDA6 R	GATCG*CCCCGGG*TTAATTTTTATTGGTCTTATAGCAATG	CDA6	
ORF-CDA7 F	ATGCG*GGATCC*ATGGCGGCACAAGATAAGTACAA	CDA7	
ORF-CDA7 R	GATCGC*CCCGGG*CTATTCAGTAATAAACTTTTTTTGGAG	CDA7	
ORF-CDA8 F	ATGCG*GGATCC*ATGGCACAGCCCATGCGCT	CDA8	
ORF-CDA8 R	GATCGC*CCCGGG*TTATTTGCAATGTAAGACCCTGAAA	CDA8	
ORF-CDA9 F	ATGCG*GGATCC*ATGGCACAACCCCCAAACC	CDA9	
ORF-CDA9 R	GATCGC*CCCGGG*CTACTGGCAATGTAAGACACGT	CDA9	
miR-s	GA**TTGACGCCTAAGAAGATCCGT** TCTCTCTTTTGTATTCC [b]	miRNA319a	
miR-a	GA**ACGGATCTTCTTAGGCGTCAA**T CAAAGAGAATCAATGA [b]	miRNA319a	Synthesis of amiR
miR*s	GA**ACAGATCTTCTTACGCGTCAT** TCACAGGTCGTGATATG [b]	miRNA319a	
miR*a	GA**ATGACGCGTAAGAAGATCTGT** TCTACATATATATTCC T [b]	miRNA319a	
OligoA	CTGCAAGGCGATTAAGTTGGGTAACG	pBlueScript SK	
OligoB	GCGGATAACAATTTCACACAGGAAACAG	pBlueScript SK	
pACN-F	TAGCTGAACATCTATATAAGG	pACN	Cloning of amiR under *A. nidulans* ethanol regulon
pACN-R	CAGAAATTATATGATAATCATCG	pACN	
pSRN-F	AAATATAGCGCGCAAACTAG	pSRN-bin	
pSRN-R	AAGGCGATTAAGTTGGGTAA	pSRN-bin	
HCa8Fdeg	ATAAAACYTYAYYAAAATAY	CaMV	3D-PCR
HCa8Rdeg	GCTTYRTAAYATYTAARATT	CaMV	
qCDA1-F	GCGGCGTTGGTTGATTATGT	*At*-CDA1	RT-qPCR quantification

qCDA1-R	CCTCTCGTATCCTCCTCCACC	*At*-CDA1	
T7-CDA1F	TAATACGACTCACTATAGGATGGATAAGCCAAGCTTCGTAATC	*At*-CDA1	RT-qPCR Standard RNA synthesis.

GenBank accession numbers: *AtCDA1*: At2g19570; *AtCDA2*: At4g29570; *AtCDA3*: At4g29580; *AtCDA4*: At4g29600; *AtCDA5*: At4g29610; *AtCDA6*: At4g29620; *AtCDA7*: At4g29630; *AtCDA8*: At4g29640; *AtCDA9*: At4g29650; CaMV: M10376. Sequences of amiR3-1-9, and of the cloning vectors pACN and pSRN-ACN are available upon request.

[a]Restriction sites are in italics

[b]21-mer sequence designed using WMD web tool for design of amiRs is in bold; WMD is available at http://wmd.weigelworld.org, and the protocols the synthesis of amiRs are thoroughly described in ref. 48.
