# Supplementary material for: A putative antiviral role of plant cytidine deaminases
Source: F1000Res. 2017 Jun 15;6:622. Originally published 2017 May 3. [Version 2] doi: 10.12688/f1000research.11111.2 (PMC5461918; doi:10.12688/f1000research.11111.2)
Supplement: Peer review history

# Peer review: Referee response for version 1

**DOI:** 10.5256/f1000research.11987.r22486

**Published:** 2017-06-02

**Authors:** Israel Pagán

**Affiliations:** 1 Center for Biotechnology and Plant Genomics (CBGP); E.T.S.I. Agrónomos ( Higher Technical School of Agricultural Engineering), Technical University of Madrid, Madrid, Spain

Martín *et al.* present an interesting work on the role of plant cytidine deaminases (CDA) as a defense mechanism against virus infection through the increase of mutational load in the viral genome during replication. CDAs are known to increase the frequency of G to A transitions. Although such mutational load has been shown to be a effective defense mechanism against some animal viruses (mainly retroviruses), this paper shows for the first time evidence that support a similar role in plants against a plant pararetrovirus. As such, I consider the paper scientifically sound.

 I find the paper well written and easy to read, and I would like to acknowledge the effort made by the authors on this aspect. The methodology is well described and all the information necessary to understand the experiments is provided. On this sense, I would just suggest adding complementary information on the number of leaves from the *N. bigelovii* agroinfiltrated with each *At*CDA. This would help to understand the degree of biological variation considered in the study.

 The main conclusion of the manuscript is that overexpression of *At*CDA leads to a decrease of viral load. I think that this conclusion is robustly supported by the data presented in the result section, and statistics are flawlessly performed and described as is the rule in the work from Prof. Elena´s group. A second main conclusion of this work is that higher viral load may be associated with the trend towards reduced frequency of G to A transitions in plants with silenced AtCDA. The authors are careful on drawing conclusions from this observation, given that the observed trend is not statistically significant. I was wondering whether the effect of the bias in G to A transitions might not be quantitative but rather qualitative. In other words, it might be interesting some discussion about the existence of a threshold in the frequency of G to A transitions bias that may lead to the reduction in viral load.

 My last suggestion relates to the observation that mutations at position 181 accounts for most of the G to A transitions. This makes me wonder about the spatial distribution of mutations (especially G to A transitions) across the viral genome. I think that including some information on whether mutations are mainly localized in coding or non-coding regions, and on whether mutations located in coding regions results mainly in synonymous and non-synonymous changes may be a nice addition. Perhaps this information may help to understand the effects of G to A transitions in the genome “functionality”.

I have read this submission. I believe that I have an appropriate level of expertise to confirm that it is of an acceptable scientific standard.

# Response

**Published:** 2017-06-09

**Authors:** Santiago F Elena

**Affiliations:** Evolutionary Systems Virology Group, Instituto de Biologia Molecular y Celular de Plantas (CSIC), Spain

Dear Dr. Pagán,

 Thank you very much for your time in reviewing the manuscript and also for your very constructive comments.  Below we provide detailed responses to each one of them.

1.  We now mention the number of half-leafs per plant (3) that were agroinfiltrated with each one of the nine *AtCDAs*.
2. We have added a brief text to the Discussion on the possibility of whether low a threshold number of G to A transitions needs to be reached in order to have a significant effect on CaMV accumulation.
3. Please, recall that we have sequenced only a region within ORF VII, thus all mutations observed are in a coding sequence.  Nonetheless, we have added extra text to the Discussion commenting on the synonymous/nonsynonymous nature of all the observed mutations, in particular for the G to A transitions most relevant for our study.  Furthermore, Supplementary Table S1 now indicates the nonsynonymous substitutions for the case of *At*CDA1 in the agroinfiltration experiments.

# Peer review: Referee response for version 1

**DOI:** 10.5256/f1000research.11987.r22521

**Published:** 2017-05-18

**Authors:** Pedro Gomez

**Affiliations:** 1 Department of Stress Biology and Plant Pathology, CEBAS - CSIC (Centre for Applied Soil Science and Biology of the Segura) - (Spanish National Research Council), Murcia, Spain

Comments of the manuscript entitled **"** **A putative antiviral role of plant cytidine deaminases** **”**.

 This manuscript reports how plant cytidine deaminases, particularly *At*CDA1, might contribute to the deamination of Cauliflower mosaic virus (CaMV) genome, and hence, affect its viral accumulation in plants. The work has merit and seems to be a good contribution. Whereas this potential antiviral response has been assessed in human and animals, virtually nothing is known about this mutagenic activity in plants.

 The experimental methods are overall solid, and the manuscript is very well-written, clear and easy to follow. 

 The authors first examined which *At*CDA proteins encoded by *Arabidopsis thaliana* have an effect on the CaMV mutational spectrum by performing an *At*CDA overexpression in *Nicotiana bigelovii*. While results are consistent with the expected AtCDA mutagenic activity, I would suggest to them to describe the reasoning behind performing it in *N. bigelovii *plants in order to clarify whether there would be any potential host AtCDAs background-noise effect that could affect or not the transient genes activity results. It may not matter, but I have not found the answers to this question within the text. If that is so, I am guessing that results from the mutant analysis spectra could even improve by providing strong results from the AtCDA1 analysis or even some differences to the other AtCDA genes could be found, as consequence of buffering those effects from negative control samples.

 Secondly, they sought to evaluate the effect of suppressing AtCDAs in transgenic A. thaliana plants on the accumulation and mutant spectrum of CaMV. Here, I am a bit concerned whether the general claim that the authors are making with this study is properly warranted. Considering that all results of this section are only based on the AtCDA1, this seems to overstate the final conclusion and perhaps this can be slightly tempered. I would recommend either to moderate this conclusion (and title) to only the atCDA1 results or to show evidence of the CaMV load reduction when suppressing the expression of the other AtCDAs. This should then be accompanied by the full description of primers and expression patterns of AtCDA2-8 mRNA analysis in the methods section, in addition to inclusion of statistics data of the mutant spectrum in the results section. This could also increase the appeal of the manuscript.

 In this sense, thinking about the general-nature of the findings, it would be very interesting and nice to read any thoughts/perspectives (in the discussion section) about this cytidine deaminase mutagenic activity in some other plant viruses (i.e. RNA virus), which could be infecting through different replicating strategies.

 Specific minor comments:

 - Please, double check this % … 471.43% increase in G to A transitions

 - Colouring treatments of the Fig 3 is a bit confusing. Please keep that as previous figures. 

 - Table S1: Please, describe that G to A substitutions detected here are shaded in the table.

I have read this submission. I believe that I have an appropriate level of expertise to confirm that it is of an acceptable scientific standard.

# Response

**Published:** 2017-06-09

**Authors:** Santiago F Elena

**Affiliations:** Evolutionary Systems Virology Group, Instituto de Biologia Molecular y Celular de Plantas (CSIC), Spain

Dear Dr. Gómez,

Thank you very much for your time in reviewing the manuscript and also for your very constructive comments.  Below we provide detailed responses to each one of them.

1. We justify the choice of *N. bigelovii* for our agroinfiltration experiments.  Basically, it was a practical choice: the clone of CaMV used in this study does not infect *N. tabacum* nor *N. benthamiana* efficiently and we needed a plant with large enough leaves to be agroinfiltrated.
2. It is true that our results only provide suggestion that *AtCDA1* may be involved in C deamination of CaMV genome.  We have edited the text to avoid making any unsubstantiated claim.  We have also added a paragraph in the Discussion putting our results in the context of recent findings that suggest that only *At*CDA1 may be relevant for the homeostasis of pyrimidines while the other eight members of the gene family may be pseudogenes.
3. We did not quantified the expression levels of *At*CDAs 2 – 9, since we decided to focus our attention in *At*CDA1 after observing that the expected bias in mutation spectrum was only found in this case.
4. We have added a new paragraph to the Discussion on the potential antiviral role of plant CDAs for other viruses.  Unfortunately, possible evidences are only limited to one Potyvirus.
5. The three specific minor comments have been considered: the percentage was correct, coloring in Fig. 3 is right and the legend of Supplementary Tables S1 and S2 have been modified to indicate that G to A transitions are shaded in grey.
